# The Role of Glucose Transporters in Oral Squamous Cell Carcinoma

**DOI:** 10.3390/biom11081070

**Published:** 2021-07-21

**Authors:** Heinrich Botha, Camile S. Farah, Kendrick Koo, Nicola Cirillo, Michael McCullough, Rita Paolini, Antonio Celentano

**Affiliations:** 1 Melbourne Dental School, The University of Melbourne, 720 Swanston Street, Carlton, VIC 3053, Australia; hbotha@student.unimelb.edu.au (H.B.); kendrick.koo@unimelb.edu.au (K.K.); nicola.cirillo@unimelb.edu.au (N.C.); m.mccullough@unimelb.edu.au (M.M.); rita.paolini@unimelb.edu.au (R.P.); 2 Australian Centre for Oral Oncology Research & Education, Perth, WA 6009, Australia; camile@oralmedpath.com.au; 3 Oral, Maxillofacial and Dental Surgery, Fiona Stanley Hospital, Murdoch, WA 6150, Australia; 4 Anatomical Pathology, Australian Clinical Labs, Subiaco, WA 6008, Australia

**Keywords:** glucose transporter, GLUT, SGLT, oral squamous cell carcinoma, oral cancer, prognosis, treatment resistance

## Abstract

Oral squamous cell carcinoma (OSCC) is a prevalent malignancy associated with a poor prognosis. The Warburg effect can be observed in OSCCs, with tumours requiring a robust glucose supply. Glucose transporters (GLUTs) and sodium-glucose co-transporters (SGLTs) are overexpressed in multiple malignancies, and are correlated with treatment resistance, clinical factors, and poor overall survival (OS). We conducted a systematic review to evaluate the differences in GLUT/SGLT expression between OSCC and normal oral keratinocytes (NOK), as well as their role in the pathophysiology and prognosis of OSCC. A total of 85 studies were included after screening 781 papers. GLUT-1 is regularly expressed in OSCC and was found to be overexpressed in comparison to NOK, with high expression correlated to tumour stage, treatment resistance, and poor prognosis. No clear association was found between GLUT-1 and tumour grade, metastasis, and fluorodeoxyglucose (FDG) uptake. GLUT-3 was less thoroughly studied but could be detected in most samples and is generally overexpressed compared to NOK. GLUT-3 negatively correlated with overall survival (OS), but there was insufficient data for correlations with other clinical factors. Expression of GLUT-2/GLUT-4/GLUT-8/GLUT-13/SGLT-1/SGLT-2 was only evaluated in a small number of studies with no significant differences detected. GLUTs 7 and 14 have never been evaluated in OSCC. In conclusion, the data demonstrates that GLUT-1 and GLUT-3 have a role in the pathophysiology of OSCC and represent valuable biomarkers to aid OSCC diagnosis and prognostication. Other GLUTs are comparatively understudied and should be further analysed because they may hold promise to improve patient care.

## 1. Introduction

Glucose transporters (GLUTs) are part of the Major Facilitator Superfamily of solute carriers which number over 400 in humans [1]. They facilitate the diffusion of soluble ions, nutrients, and other metabolites across the hydrophobic cell membrane [2]. The GLUT family of transporters consists of 14 trans-membrane proteins coded by *SLC2A* genes and are primarily known for the transport of glucose and other hexose molecules such as fructose [3]. They also transport uric acid [4], ascorbate [5,6], glucosamine [7], myo-inositol [8] and many other substrates. GLUTs vary widely in their tissue distribution, substrate affinity, and turnover rates.

Oral squamous cell carcinoma (OSCC) is the ninth most prevalent cancer globally, with 354,864 new cases diagnosed and 177,384 deaths in 2018 [9]. Despite advancements in treatment, the five-year overall survival of approximately 50% has remained unchanged in the past few decades [10]. Dysregulation of metabolic pathways is a key hallmark of cancer [11], and OSCCs rely upon aerobic glycolysis as their primary method of ATP production [12,13,14]. Glycolytic enzymes are upregulated in a wide range of cancer cells [15,16,17] and GLUTs are often overexpressed [18]. This ensures a robust supply of glucose to fuel rapid growth and proliferation [18,19].

GLUT expression has previously been correlated with poor prognosis in several cancers including OSCC [20,21,22,23]. GLUT-1 expression has been associated with both chemo-resistance and radio-resistance in multiple malignancies including in OSCC [21,24,25,26,27]. These associations may be due to the effects of upregulated DNA repair mechanisms [28] and the expression of anti-apoptotic genes [29] as a result of increased glycolysis. Greater GLUT expression has been observed in cancers of advanced stages and higher tumour grades [30,31,32,33]. Despite the large number of publications investigating this important transporter family in OSCC, they have never been systematically evaluated.

In the present study, we have conducted a systematic review into the role of glucose transporters in OSCC. The expression of glucose transporters in OSCC relative to normal oral keratinocytes (NOK) was compared, and the impact of these alterations on clinical factors, such as prognosis, clinical staging, grade, differentiation, metastasis, risk factors and fluorodeoxyglucose (FDG) uptake, was explored.

## 2. Materials and Methods

### 2.1. Research Questions

Specific questions addressed in this study included:

1. Are there differences in glucose transporter expression between OSCC and NOK?
2. Is glucose transporter expression related to the rate of disease progression, clinical factors, patient prognosis, treatment resistance, and the hallmarks of cancer?
3. Are there effective treatment strategies targeting glucose transport in OSCC?

### 2.2. Reporting Methodology

The Preferred Reporting Items for Systematic Reviews and Meta-Analyses (PRISMA) [34] chart was followed during the data collection and screening phases of this review.

### 2.3. Literature Search

On the 22 July 2020, a comprehensive online search of the OVID Medline and Web of Science (WOS) databases was conducted by two independent reviewers (HB, AC). No limits were placed on either database search. ‘Keyword’ search was used on Medline and ‘Topic’ search was used on WOS. The search terms according to the syntax rules of each database are displayed in Supplementary Materials 1.

### 2.4. Data Collection

Two independent, blinded reviewers (HB, AC) were involved in the data collection and screening phases. After conducting the search, all citations retrieved from Medline and WOS were exported to Endnote X9 (Clarivate Analytics, Philadelphia, PA, USA), where duplicates were first removed by the software and then manually by the reviewers. 

### 2.5. Screening

Studies were then screened using our inclusion and exclusion criteria first by their title, then abstract, and finally by full text. 

Inclusion criteria:

1. Glucose transporters or sodium-glucose co-transporters.
2. Research on OSCC or healthy oral squamous cells.

Exclusion criteria:

1. Potentially malignant lesions of the oral cavity.
2. Non-English research.
3. Case reports, case series, letters, conference abstracts, meta-analyses, reviews, and retracted studies.
4. Non-peer-reviewed literature.

Any studies that yielded disagreements between reviewers in the title and abstract phases were included in the next round of screening. Disagreements in the full-text screening phase were settled by discussion until consensus was reached.

### 2.6. Data Extraction

Relevant data from the 85 papers was extracted to an Excel (Microsoft Excel 2011, Redmond, WA, USA) data collection spreadsheet. Information collected included: biomarkers, model system, GLUT assay methodology, human specimen type, anatomical location of samples, number of samples/cell lines/mice, clinical stage, metastasis, grade, risk factors, age, sex, treatment, GLUT inhibitors, follow-up, and prognostic value. Data extraction was independently checked by two reviewers (AC and CSF) to ensure consistency and quality.

### 2.7. Risk of Bias Analysis

Risk of bias for the included prognostic studies that evaluated the prognostic role of glucose transporters was assessed using the “Quality in Prognosis Studies” (QUIPS) tool [35], which evaluates the following six domains: “study participation”, “study attrition”, “prognostic factor measurement”, “outcome measurement”, “study confounding”, and “statistical analysis and reporting”.

## 3. Results

### 3.1. Study Selection and Screening

We retrieved 552 and 646 citations from Medline and WOS, respectively, yielding a total of 1198 papers. After removing a total of 417 duplicates via software and manually, 781 unique records were left for screening. 

**Figure 1:**
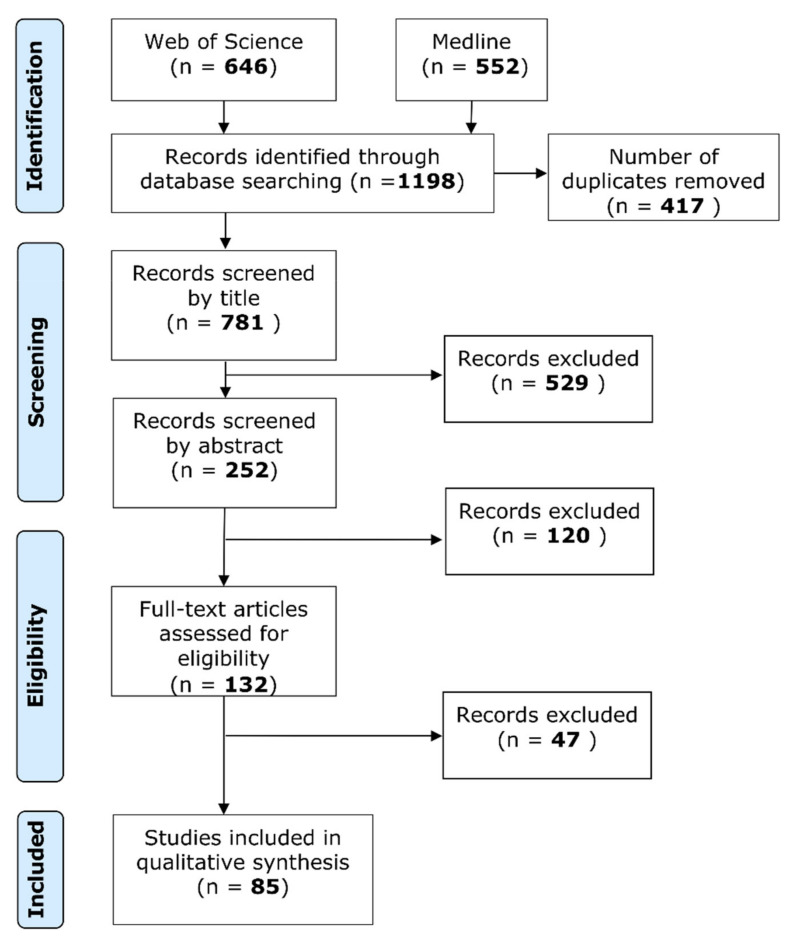
PRISMA flow chart of the systematic review.

After screening by title, 252 studies were included. Cohen’s Kappa coefficient was 0.93 (95% CI: 0.91–0.95) and inter-rater agreement was 93.47%. The subsequent step of screening by both title and abstract resulted in 132 studies being included, with a Kappa coefficient of 0.89 (95% CI: 0.85–0.93) and inter-rater agreement of 89.68%. Full-text screening of the included studies resulted in 85 being selected for review, with a Kappa coefficient of 0.87 (95% CI: 0.81–0.93) and inter-rater agreement of 87.88%. Studies in the full-text phase were primarily excluded due to an inability to distinguish OSCC data from other head and neck squamous cell carcinomas (HNSCC), and occasionally the lack of reporting of GLUT expression data. The literature search and screening process is summarised in Figure 1.

### 3.2. Quality in Prognostic Studies (QUIPS) Analysis

**Figure 2:**
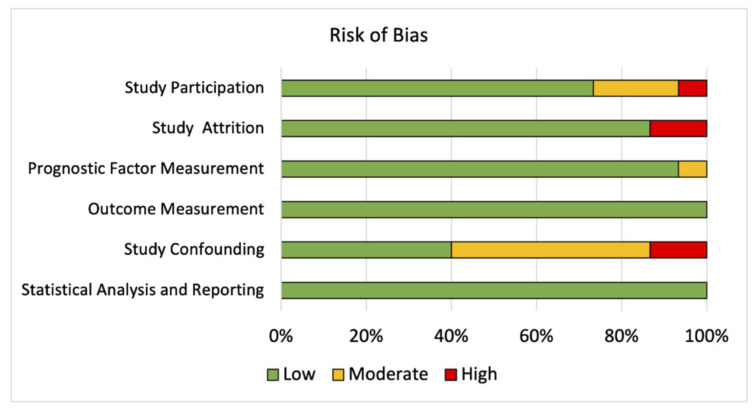
Summarized risk of bias in the 15 included prognostic studies according to the Quality in Prognosis Studies (QUIPS) criteria [35]. Individual ratings are displayed in Supplementary Material 2.

A QUIPS analysis was performed on all prognostic studies to measure the risk of bias [35]. Low risk of bias was observed in “study participation”, “study attrition”, “prognostic factor measurement”, “outcome measurement”, “study confounding”, and “statistical analysis and reporting” (73%, 87%, 93%, 100%, 40%, and 100% of included studies, respectively), while moderate risk of bias in “study participation” was found in 20% of studies and in “prognostic factor measurement”, and “study confounding” in 7% and 47% of the studies, respectively. The percentage of studies with a high risk of bias was relatively low and varied between 7% and 13% across three different domains (Figure 2). The full QUIPS analysis can be found in Supplementary Materials 2.

### 3.3. Glucose Transporter Expression in OSCC and NOK Normal Oral Keratinocytes

The studies included in this systematic review encompass a variety of samples, including cell lines and patient derived specimens. In addition, the GLUT family members were assayed by a range of laboratory techniques. Therefore, the results have been grouped by GLUT member and further stratified by specimen and assay type. In line with established conventions, gene names in italics (e.g., *SLC2A1*) are used to describe studies reporting mRNA transcripts, and protein names (e.g., GLUT-1) are used where protein expression is reported.

### 3.4. GLUT-1 (SLC2A1)

#### 3.4.1. Cell Lines

Findings for *SLC2A1* in six cell line studies [36,37,38,39,40,41] and GLUT-1 protein expression in seven cell line studies [37,40,41,42,43,44,45] were evaluated and summarised in Table 1. The majority of the reviewed studies identified consistent overexpression of *SLC2A1* and GLUT-1 in OSCC cell lines. 

#### 3.4.2. Patient Samples (mRNA)

Seven studies evaluated *SLC2A1* expression in patient-derived tissue [37,43,46,47,48,49,50]. These findings are summarised in Table 2. *SLC2A1* transcripts were generally found to be increased in OSCC samples when compared to their normal tissue counterparts. 

#### 3.4.3. Patient Samples (Protein)

GLUT-1 expression at the protein level in patient samples was evaluated in nearly half of the included studies (32/85) [23,45,51,52,53,54,55,56,57,58,59,60,61,62,63,64,65,66,67,68,69,70,71,72,73,74,75,76,77,78,79,80]. These findings are summarised in Supplementary Table S1. Whilst there were differences in methodology and staining thresholds for classification, the majority of patient samples were found to be GLUT-1 positive (1380/1709, 80.7%). There were 11 studies (579 patients) where all samples (100%) demonstrated positive staining for GLUT-1 [27,43,51,52,56,59,61,63,71,72,76,80] and a further 11 studies reported GLUT-1 expression in 70%–98% of tumour samples [23,53,55,60,64,73,74,75,77,78,79].

There was, however, significant variation in the percentage of cells staining positive for GLUT-1 in individual tumours: median staining of 60% (0–90%) was observed by Gronroos and colleagues [70], 26% (0–60%) by Choi et al. [65], and 65.60% (±25.67) by Azad et al. [61]. 

Staining intensity was reported by numerous authors, and although the vast majority of tumours fell into the “moderate” to “high” ranges, there were drastically different findings, with intense staining (IHC score ≥ 3) of GLUT-1 found to range from 0.3% to 63% in eight studies [52,54,56,63,64,72,73,76].

**Figure 3:**
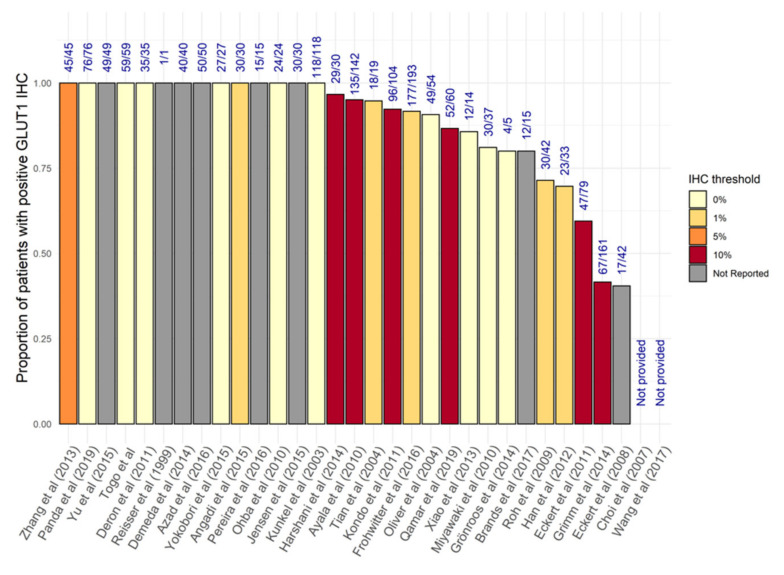
Proportion of patients with positive GLUT-1 expression in the 85 included studies according to immunohistochemical findings.

A few studies also evaluated GLUT-1 expression in normal epithelial tissues, with all showing undetectable or weak expression [53,67,68,69,74]. Where comparisons were made between tumour and normal tissues, GLUT-1 expression was significantly upregulated [23,36,37,43,45,66,69,74] and in a single study, GLUT-1 was found to be elevated in the serum of OSCC patients [43]. The percentage of tumours positive for GLUT-1 in 30 studies is reported in Figure 3 [23,45,51,52,53,54,55,56,57,58,59,60,61,62,63,64,66,67,68,70,71,72,73,74,75,76,77,78,79,80].

### 3.5. GLUT-3 (SLC2A3)

Nine studies assessed GLUT-3 expression in patient tumours, and a further two studies evaluated expression in cell lines. These findings are set out in Table 3. There were inconsistent findings for mRNA and protein expression across papers. However, most studies found upregulated GLUT-3 in a subset of samples. 

### 3.6. GLUT-4 (SLC2A4)

Seven studies evaluated the expression of GLUT-4 in a variety of tissues, and their findings are set out in Table 4. Detection of GLUT-4 and its mRNA were frequently seen in tumours and OSCC cell lines but was completely absent in healthy keratinocytes. However, the sample sizes were very small, and the number of studies is limited.

### 3.7. Other GLUT Family Members

A handful of studies investigated GLUT-2, GLUT-8, GLUT-13 and SGLTs, but the number of reports was too small to draw any meaningful conclusions for these proteins. A summary of their findings is set out in Supplementary Table S2.

### 3.8. GLUTs and Clinicopathologic Characteristics

Two out of four studies reported an association between tobacco smoking and GLUT expression. GLUT-1 overexpression was significantly correlated with smoking and a larger percentage of cells stained positive for GLUT-1 in smokers (79.2% vs 52%) in a cohort of 50 patients by Azad et al. [61]. Brands et al. determined that smoking increased the probability of tumour GLUT-1 positivity by three-fold [74]. Estilo et al. found that 30.6% of patients had high levels of GLUT-3 expressed, compared to normal tissue [81]. In contrast, Qamar found in a cohort of 60 patients that GLUT-1 was only positive in 10% of smokers but was expressed in 82.7% of non-smokers [64]. Similarly, Choi et al. found that smoking status was not associated with GLUT-1 expression.

A single study investigated the effect of alcohol consumption, finding an association with GLUT-1 tumour positivity and the percentage of cells stained [23]. A study on a single HPV-positive cell line, 147T, had higher levels of GLUT-1 expression than the HPV-negative cell line Cal33 [86].

Seven studies reported significant correlation between GLUT-1 overexpression and higher tumour grade [23,51,61,66,67,87,88]. Panda et al. found intense GLUT-1 expression in 23.3% of Grade 1, 69.6% of Grade 2, and 100% of Grade 3 OSCCs (r = 0.885, *p* = 0.001) [51]. Similarly, a strong correlation was also found by Azad et al. based on Bryne’s grading system (*p* < 0.001) [61]. Vasconcelos et al. observed that 90.7% of high grade tumours showed strong staining, and only 35.7% of low grade tumours had strong staining [87]. However, there were seven additional studies where no correlations were observed [48,54,59,60,62,65,89].

Fourteen studies reported on metastasis, with eight finding correlations with GLUT-1 [53,61,65,68,75,83,89,90] and six finding no association [23,48,54,62,72,87]. A large study with 104 patients reported that only 6/26 patients with metastasis were GLUT-1 positive, <30% (*p* = 0.016) [53]. However, Nakazato et al. reported in a cohort of 110 patients that all of those with nodal metastasis had high GLUT-1 expression [48]. 

Five studies observed a correlation between clinical TNM stage (tumour, nodes, and metastasis) and GLUT-1 expression [61,66,87,89,90]. Vasconcelos et al. found that strong GLUT-1 staining increased with TNM stage (42.9% of stage 1, 70% of stage 2, 90.9% of stage 3, and 100% of stage 4) in 57 patients [87]. The correlation was highly significant (*p* = 0.002). A similarly sized cohort of 50 patients found an even stronger correlation (*p* < 0.001) [61]. There was only one paper that found no correlation between GLUT-1 and clinical stage [74].

The body of evidence for GLUT-3 and GLUT-4 was significantly smaller than that for GLUT-1. There was no correlation between GLUT-3 and tumour grade [60] but GLUT-3 expression was consistently overexpressed in the deep invasive front regardless of the presence of metastasis *p* = 0.482, early vs. late stages (*p* = 0.892), or low vs. high tumour grades (*p* = 0.384) [59]. Feitosa and colleagues reported no correlation between expression and age or gender of the patient and GLUT-3 and GLUT-4 expression [82], although most samples with GLUT-4 cell positivity over 80% were located at the floor of the mouth or base of the tongue [82].

No studies exploring correlations to other GLUT family members could be identified. 

### 3.9. GLUTs and Tumour FDG Uptake

Ten studies assessed the relationship between GLUT expression and FDG uptake in pre-operative PET scans. Half of these found a correlation with GLUT-1 [46,56,77,78,91], whilst the others did not [60,63,80,88,92]. Two xenograft mouse studies undertaken by Wilson et al. and Silén et al. examined this relationship and found no correlation [93,94]. GLUT-3 was analysed in two patient studies and no correlations were found with FDG uptake [46,60].

### 3.10. GLUT Inhibitors

Kraus et al. tested the GLUT-1 inhibitors STF-31, WZB117, and Fasentin on BHY and HN OSCC cell lines [95]. All inhibitors reduced the viability of both cell lines significantly, but STF-31 was the most effective. The same authors later tested WZB117 and Fasentin on BHY and HN cell lines, this time measuring glucose uptake. WZB117 was significantly more effective, reducing glucose uptake by approximately 90% in both cell lines, compared to 10%–35% for Fasentin.

### 3.11. GLUTs and Treatment Response

The impact of GLUT-1 expression on chemotherapy and radiation therapy responses have been evaluated. Pre-clinical studies suggest improved radiation and chemotherapy responses by decreasing GLUT-1 expression: 2-deoxy-d-glucose (2-DG) was found to significantly decrease GLUT-1 expression in both normal and OSCC cell lines, and concurrent administration increased the effectiveness of radiation therapy in the SAS but not HSC-3 and HSC-4 cell lines [42]; the rate of apoptosis of CAL27 cells in response to cisplatin was increased by an anti-GLUT-1 antibody [40]; GLUT-1 overexpressing cell lines were more resistant to cisplatin, and sh-RNA silencing of GLUT-1 significantly increased rates of apoptosis in CAL27 and SCC25 cells. In a cisplatin-resistant model of CAL27 and SCC25 OSCC cells, SLC2A1 levels were 1.53 and 1.37-fold higher, respectively, compared to their parental cell lines [41]. In a xenograft mouse model, radiation treatment resulted in an initial reduction in mean GLUT-1 immunostaining positivity of 41.9% to 30.7% over 12 days, although this did rebound upon tumour regrowth.

Clinical studies were unfortunately less conclusive, and it remains unclear if GLUT-1 would serve as a useful biomarker for treatment response. In a cohort of 60 patients, Choi et al. found that higher GLUT-1 expression was more likely to require adjuvant radiotherapy [65]. Kunkel et al. found that patients demonstrating a response to neoadjuvant radiation therapy (36Gy/18 fractions) had significantly lower GLUT-1 expression and percentage cell positivity, and patients with above median GLUT-1 expression demonstrated improved survival [27]. However, Miyawaki et al. found no association between GLUT-1 expression and histological treatment response in tumours of patients who received neoadjuvant chemoradiotherapy [78].

EGFR inhibitors (EGFRi) were assessed for their relationship with GLUT-1 in four studies. After treatment with Cetuximab, GLUT-1 mRNA in UT-SCC-14 and UT-SCC-45 cells significantly increased, but not in UT-SCC-2 [96]. However, in a similar study, Gustafsson et al. found GLUT-1 expression increased in both UT-SCC-14 and UT-SCC-2 cell lines [97]. Furthermore, CAL27 xenograft tumours that were designed to be EGFRi resistant had significantly greater GLUT-1 expression (*p* < 0.05) [98]. Conversely, erlotinib, another EGFRi, had no effect on GLUT-1 expression in CAL33 and CAL166 cell lines or xenograft tumours [44].

GLUT-1 expression and its association with prognosis were analysed in patient tumours in 13 studies. Five studies found no correlation with OS [48,52,53,73,77], while eight studies [23,27,54,62,65,68,72,80] showed that GLUT-1 had a significant negative correlation with OS. Eckert et al. reported that patients with low GLUT-1 expression had a median survival of 51.0 months compared to 34.3 months (*p* = 0.004) for patients with high expression [54]. Another study by the same group found that for patients with negative or weakly stained tumours, five-year survival was 74% but 24% for those with moderate–strong staining tumours [62]. GLUT-1 was found to be an independent prognostic factor after accounting for clinical factors such as tumour size, T stage, and lymph node status [62]. GLUT-1 was determined to be an independent prognostic factor in three additional studies [23,72,80]. In terms of time to disease relapse or disease-free survival, GLUT-1 was found not to be significantly associated, despite showing a correlation with OS [65]. Han et al. found no correlation with disease-free survival [77]. Kunkel et al. found that the percentage of cells positive for GLUT-1 in a tumour was predictive of the survival of patients undergoing preoperative radiation therapy [27]. In another study by Kunkel et al., the percentage of positive cells was more predictive of prognosis than the intensity of GLUT-1 staining [80]. Those with a cell positivity of <50% had a median survival of 138 months, compared to 60 months for those with >50% cell positivity (*p* = 0.0034). Finally, an analysis of GLUT-1 protein in the serum of OSCC patients found that OS was lower in those with high levels [43].

GLUT-3 was examined in relation to prognosis in only two studies. Ayala et al. showed that GLUT-3 was strongly associated with lower OS (*p* = 0.002), worse disease-free survival (*p* = 0.021) and a greater risk of tumour recurrence [23]. Multivariate analysis found that GLUT-3 was an independent prognostic factor in overall survival. In a study of 49 patients, Estilo and colleagues similarly showed a prognostic value for GLUT-3 as it relates to relapse-free survival (*p* = 0.002), disease-free survival (*p* = 0.049), and OS (*p* = 0.003) [81].

## 4. Discussion

This systematic review set out to elucidate the role of GLUTs in the pathophysiology of OSCC and in determining patient outcomes. We hypothesised that there would likely be aberrations in GLUT expression to meet a high demand for glucose due to upregulated glycolysis.

Our review found that GLUT-1 protein and mRNA were consistently overexpressed, with overexpression relative to NOK cell lines in 12 of 13 cell-line studies [36,37,38,39,40,41,42,43,44,45]. Similarly, mRNA was highly expressed in all patient tumour studies [37,46,47,48,49,50] and mostly absent in adjacent healthy tissues. Protein expression of GLUT-1 in patients was the most studied marker [23,27,37,39,43,45,51,52,53,54,55,56,57,58,59,60,61,62,63,64,65,66,67,68,69,70,71,72,73,74,75,76,77,78,79,80,94,99]. Not all of the findings of these papers converged, however the majority of papers reported that most samples were positive for GLUT-1. Of note, all studies in NOKs showed absent or weak expression, and GLUT-1 was consistently overexpressed in OSCC cells compared to healthy adjacent tissue [53,67,68,69,74]. GLUT-1 was generally upregulated in OSCC and may play a vital role in glucose homeostasis. GLUT-1 is known to be the transporter that facilitates basal uptake due to its high affinity for glucose (Km = 2 mM) [100]. This also reflects the findings in studies of other cancer types including breast [101,102], colorectal [22], prostate [103], and non-small cell lung cancer [104], where GLUT-1 is frequently overexpressed. The second most-studied transporter was GLUT-3; although there is only limited research. mRNA studies found mixed results, where GLUT-3 was expressed in cell lines [38] and frequently overexpressed in OSCC tumours compared to adjacent healthy tissues [46,81], with some exceptions [48]. Immunohistochemistry analyses pointed towards some GLUT-3 protein expression with an average positivity rate of 45.3% (range: 0–100%) of all patient tumours [23,46,59,60,82]. GLUT-4 protein expression in OSCC cell lines appeared quite prevalent [38,85], however patient studies showed mixed results with very small sample sizes. GLUT-4 was not expressed in NOKs [84]. In relation to SGLT-1, SGLT-2, GLUT-2, GLUT-8 and GLUT-13, few conclusions can be drawn based upon a limited number of studies.

In terms of clinical factors and patient outcomes, GLUT-1 was repeatedly implicated. The majority of prognostic studies correlated high GLUT-1 expression with poor overall survival [23,27,54,62,65,68,72,80]. Furthermore, in four of these studies, GLUT-1 was found to be an independent prognostic factor, accounting for clinical factors such as stage, grade, tumour size, and lymph node status [23,62,72,80]. Surprisingly, GLUT-1 did not show a correlation with disease-free survival [65,77]. The data on GLUT-3 was limited, however supported GLUT-3 expression being significantly associated with reduced time to relapse, disease-free survival, and OS [23,81]. The data on clinical staging overwhelmingly pointed to GLUT-1 expression being positively correlated with stage [61,66,87,89,90], with only a single contradictory study [74]. This study only included 15 patients, while the studies that found a positive correlation amounted to 231 patients in total [74]. For grading and tumour differentiation the role of GLUTs is unclear. Half of the 14 studies undertaken observed a positive correlation for GLUT-1 with grading and tumour differentiation, while the rest found no association. Similarly, for metastasis, it was challenging to draw any conclusions since there was not a significant consensus between studies. This is surprising, since a recent systematic review and meta-analysis found GLUT-1 to correlate with both grade and lymph node metastasis in many cancer types [105]. The findings on the association between tumour fluorodeoxyglucose (FDG) uptake in patients and GLUT-1 expression were similar, in that there was significant disagreement between studies. The combined cohorts for the five studies finding a correlation were 172 versus 207 in the five studies showing no relationship. This indicates that, in OSCC, GLUT-1expression may not be the limiting factor for glucose metabolism.

Risk factors were under-studied for association with GLUTs. Smoking was found to correlate with GLUT-1 expression in three studies, but was contradicted by a single study showing the GLUT-1 was higher in non-smokers. A single in vivo study on patient alcohol consumption and another on HPV positive cell lines found both risk factors to correlate with higher GLUT-1 expression.

Inhibition of GLUTs as a treatment strategy has not been extensively studied. However, two studies by the same authors were conducted using GLUT-1 inhibitors (STF-31, WZB117, and Fasentin) which found that cell viability [95] and glucose uptake [106] were significantly reduced in two cell lines. Further research with GLUT inhibition is needed.

## 5. Conclusions

With the exception of GLUT-1 and possibly GLUT-3, glucose transporters are relatively understudied in OSCC as well as healthy oral keratinocytes. There is room for further research to obtain a clearer picture of which GLUTs are most important in OSCC metabolism. In addition, it may be of great value to clinicians to better understand any association these proteins may have with disease progression, treatment resistance, and prognosis, to better inform treatment decisions.

## Figures and Tables

**Table 1:** GLUT1 in cell lines.

Author	Modality	Cell Lines	Findings
Grimm et al. (2014) [36]	mRNA	BICR3, BICR56	BICR3 and BICR56 were 22.4-fold and 25.3-fold increase in SLC2A1 expression, respectively, compared to the NOK cell line, HOK
Chen et al. (2019) [37]	mRNA	Tca8113, CAL27	GLUT-1 expression was significantly increased in Tca8113 and CAL27 cell lines compared to the NOK cell line, NK.
Fukuzumi et al. (2000) [38]	mRNA	SAS, Nakata, Ueda, KN, OSC-2, 4, 5, 6, and HSC-2	GLUT1 and GLUT3 mRNA was expressed in all cell lines but was higher in OSCC than normal epithelial cells. Expression of GLUT2 mRNA was detected in 5/9 OSCC cell lines and in both the normal epithelial cells. Expression of GLUT4 mRNA was detected in 6/9 OSCC cell lines but not in both normal epithelial cell lines used.
Wang et al. (2013) [40]	mRNA	Cal27	qPCR showed SLC2A mRNA expression.
Li et al. (2015) [41]	mRNA	CAL27, SCC25 and cisplatin resistant variants of both cell lines.	Cisplatin resistance resulted in greater SLC2A expression.
Chen et al. (2019) [37]	protein	Tca8113, CAL27	GLUT-1 strongly expressed in OSCC vs NOK
Wang et al. (2013) [40]	protein	Cal27	Western blot showed GLUT-1 expression.
Li et al. (2015) [41]	protein	CAL27, SCC25 and cisplatin resistant variants of both cell lines.	GLUT-1 expression in Cisplatin resistant variants was 1.37-1.53 times greater compared to parental cell lines.
Kawata et al. (2017) [42]	protein	SAS, HSC-4, HSC-3	GLUT-1 strongly expressed in OSCC vs NOK
Xu et al. (2018) [43]	protein	SCC09, SCC15, SCC25	GLUT-1 strongly expressed in OSCC vs NOK
Vergez et al. (2010) [44]	protein	CAL33 and CAL166	GLUT-1 was expressed but not GLUT-3 and GLUT-4.
Zhang et al. (2013) [45]	protein	YD9, YD10B, YD38, IHOK	GLUT1 not detected or weakly expressed

**Table 2:** SLC2A1 expression in patients.

Author	Number of Patients	Findings
Chen et al. (2019) [37]	20	SLC2A1 expression significantly greater in OSCC tissue samples than healthy oral tissue specimens (*p* < 0.05)
Li et al. (2008) [46]	7	*SLC2A1* significantly higher in the OSCC tissue of all 7 patients compared to contralateral normal tissues (*p* < 0.0001)
Mellanen et al. (1994) [47]	5	All samples tested positive for SLC2A1 mRNA.
Nakazato et al. (2019) [48]	110	*SLC2A1* was the only SLC2A gene for which mRNA was overexpressed compared to healthy adjacent tissue (*p* = 0.005) Significant inter-patient variation for SLC2A1 expression between tissues of the same category (*p* = 0.009)
Chu et al. (2019) [49]	60	*SLC2A1* was significantly higher in the OSCC tissues of all patients compared to adjacent normal tissues.
Chen et al. (2019) [50]	52	*SLC2A1* was significantly higher in the OSCC tissues of all patients compared to adjacent normal tissues (*p* < 0.05).
Xu et al. (2018) [43]	68	GLUT-1 was significantly upregulated in tumours compared to adjacent healthy tissue. GLUT1 upregulated in OSCC patient serum compared to healthy controls.

**Table 3:** Summary of findings for GLUT-3.

Author	Tissue Type	Modality	Findings
Estilo et al. (2009) [81]	Tumour samples	mRNA	Greater than two-fold increase in SLC2A3 transcripts in 30.6% of 49 tumours compared to healthy tissues
Nakazato et al. (2019) [48]	Tumour samples	mRNA	SLC2A3 expression not significantly different compared to healthy tissues for 110 patients
Mellanen et al. (1994) [47]	Tumour samples	mRNA	SLC2A3 expressed in only 1/5 tumour samples
Li et al. (2008) [46]	Tumour samples	mRNA protein	Greater expression of SLC2A3 in tumours than contralateral normal tissue from 7 patients No GLUT-3 expression in all 7 patients
Demeda et al. (2014) [59]	Tumour samples	protein	Positive GLUT-3 IHC in 40 OSCC, but lower percentage of positive cells than for GLUT-1
Tian et al. (2004) [60]	Tumour samples	protein	16/19 tumours positive, with an average of 97.5% cell positivity Only 36.8% of tumours co-expressed GLUT-1 and GLUT-3
Feitosa et al. (2018) [82]	Tumour samples	protein	GLUT-3 expression in all 15 cases, 10%-89% of cells positive
Ayala et al. (2010) [23]	Tumour samples	protein	30 of 142 (21.1%) GLUT-3 positive No correlation between GLUT-1 and GLUT-3 expression
Jonathan et al. (2006) [83]	Tumour samples	protein	GLUT-3 staining in a median of 25.1% of 5 tumours
Fukuzumi et al. (2000) [38]	Cell lines	mRNA	Variable detection of SLC2A3 in 9 OSCC cell lines. SLC2A3 expression elevated in OSCC over normal keratinocyte cell lines
Vergez et al. (2010) [44]	Cell lines	protein	Undetectable in CAL33 and CAL166 cell lines

**Table 4:** Summary of findings for GLUT-4.

Author	Tissue Type	Modality	Findings
Mellanen et al. (1994) [47]	Tumour samples	mRNA	*SLC2A4* undetectable in 5/5 samples
Fukuzumi et al. (2000) [38]	Cell lines	mRNA	*SLC2A4* identified in cancer cell lines (6/9) but undetected in normal keratinocyte cell lines (0/2)
Reisser et al. (1999) [58]	Tumour samples	protein	GLUT-4 undetectable in 1/1 sample
Feitosa et al. (2018) [82]	Tumour samples	protein	15.2-79.9% of cells positive for GLUT-4 in 15/15 samples
Voldstedlund et al. (1997) [84]	Normal epithelium	protein	GLUT-4 undetectable in 12/12 samples
Vergez et al. (2010) [44]	Cell lines	protein	GLUT-4 undetectable in CAL33 and CAL166 cell lines
Chang et al. (2017) [85]	Cell lines	protein	GLUT-4 detected in HSC-3, HSC-M3, HSC-2, HSC-4, FaDu, Detroit-562, RPMI-650, and Ca-922 cell lines

## Supplementary Materials

The following are available online at https://www.mdpi.com/article/10.3390/biom11081070/s1, Supplementary Materials 1: search terms, Supplementary Materials 2: QUIPS studies, Table S1: GLUT-1 protein expression in patient tumours (IHC), Table S2: Summary of findings for less studied GLUT family members.
