# Supplementary material for: The Role of Glucose Transporters in Oral Squamous Cell Carcinoma
Source: Biomolecules. 2021 Jul 21;11(8):1070. doi: 10.3390/biom11081070 (PMC8392467; doi:10.3390/biom11081070)
Supplement: Supplementary file 1 [file biomolecules-11-01070-s001.zip › Supplementary Materials 1_Search Terms.pdf]

Supplementary Materials 1 – Search Terms

oral OR mouth OR lip OR palate OR buccal mucosa OR cheek OR retromolar OR floor of mouth OR gingiva OR tongue OR squamous cell

AND

cancer* OR neoplas* OR tumour* OR tumor* OR malignan* OR carcinoma* OR metastas* OR normal

AND

glucose transport proteins, facilitative OR glucose transport* OR slc2a OR solute carrier family 2 OR sodium-glucose transport protein* OR sodium glucose transporter* OR sodium-glucose transporter* OR sglt* OR slc5a OR sodium-dependent glucose cotransporter* OR solute carrier family 5 OR glut

AND

initiation OR tumorigenesis OR tumourigenesis OR carcinogenesis OR progression OR metastasis OR prognos* OR express* OR inhibit* OR treat* OR drug therapy.
