# Supplementary material for: The Role of Glucose Transporters in Oral Squamous Cell Carcinoma
Source: Biomolecules. 2021 Jul 21;11(8):1070. doi: 10.3390/biom11081070 (PMC8392467; doi:10.3390/biom11081070)
Supplement: Supplementary file 1 [file biomolecules-11-01070-s001.zip › Supplementary Materials 2_QUIPS studies.pdf]

Supplementary Materials 2 – QUIPS: studies evaluated

First Author Surname	Year	Study Participation	Study Attrition	Prognostic Factor Measurement	Outcome Measurement	Study Confounding	Statistical Analysis and Reporting
Yu	2015	LOW	LOW	LOW	LOW	MODERATE	LOW
Kondo	2011	LOW	LOW	LOW	LOW	MODERATE	LOW
Eckert	2011	LOW	HIGH	LOW	LOW	MODERATE	LOW
Eckert	2008	LOW	LOW	LOW	LOW	MODERATE	LOW
Nakazato	2019	LOW	LOW	LOW	LOW	LOW	LOW
Choi	2007	LOW	LOW	LOW	LOW	LOW	LOW
Grimm	2014	LOW	LOW	LOW	LOW	LOW	LOW
Ayala	2010	LOW	HIGH	LOW	LOW	LOW	LOW
Xu	2018	MODERATE	LOW	LOW	LOW	HIGH	LOW
Estilo	2009	LOW	LOW	LOW	LOW	LOW	LOW
Kunkel	2003	LOW	LOW	LOW	LOW	MODERATE	LOW
Ohba	2009	MODERATE	LOW	LOW	LOW	HIGH	LOW
Kunkel	2007	MODERATE	LOW	LOW	LOW	LOW	LOW
Oliver	2004	HIGH	LOW	MODERATE	LOW	MODERATE	LOW
Han	2011	LOW	LOW	LOW	LOW	MODERATE	LOW
