# Supplementary material for: The Role of Glucose Transporters in Oral Squamous Cell Carcinoma
Source: Biomolecules. 2021 Jul 21;11(8):1070. doi: 10.3390/biom11081070 (PMC8392467; doi:10.3390/biom11081070)
Supplement: Supplementary file 1 [file biomolecules-11-01070-s001.zip › Table S1 - GLUT1 protein expression in patient tumours (IHC).pdf]

Table S1. GLUT1 protein expression in patient tumours (IHC).

Author	Number of Patients	Number positive (%)	IHC cut-off	Percentage of cells stained positive	Staining Intensity	Other findings
Ayala et al (2010)	142	135 (95.07%)	>10% of cells stained moderately	<85% in 58 tumours. >85% in 77 tumours.	NR	None
Zhang et al (2013)	45	45 (100%)	>5%	"Strong expression" in 28 tumours. "weak expression" in 17 tumours.	NR	None
Panda et al (2019)	76	76 (100%)	Any visible staining.	NR	Intense: 27<br>Mild: 49	None
Yu et al (2015)	49	49 (100%)	NR	NR	NR	None
Kondo et al (2011)	104	96 (92.31%)	>10%	37%	NR	The mean positive rate for GLUT-1 in all tumour cells was 34%.<br>No GLUT1 detected in normal samples
Eckert et al (2011)	79	47 (59.49%)	>10%	NR	Weak:13<br>Moderate: 21<br>Strong: 11	GLUT-1 was expressed weakly in 13, moderately expressed in 22, and strongly expressed in 12.
Frohwitter et al (2016)	193	177 (91.71%)	≥ 1%	NR	NR	None
Togo et al	59	59 (100%)	Any membranous staining.	NR	NR	None
Deron et al (2011)	35	35 (100%)	>0%	NR	NR	None
Reisser et al (1999)	1	1 (100%)	NR	NR	NR	None
Demeda et al (2014)	40	40 (100%)	NR	NR	NR	None
Tian et al (2004)	19	18 (94.74%)	≥ 1%	Mean: 79.74%	Weak: 4<br>Moderate: 3<br>Strong: 11	Stained weakly in 4 tumours, moderately in 3, strongly in 11.
Azad et al (2016)	50	50 (100%)	NR	Mean: 65.6%	NR	Cell positivity varied between 15% to 95% between tumours (mean 65.60±25.67%).
Eckert et al (2008)	42	17 (40.48%)	NR	NR	Weak: 7<br>Moderate: 8<br>Strong: 2	Moderate to strong GLUT-1 staining: 10 patients Negative to weak GLUT-1 staining: 32 patients
Yokobori et al (2015)	27	27 (100%)	>0%	<10%: 2<br>11–25%: 8<br>26–50%: 14<br>>50%: 3	NR	IHC scoring: 2 patients with score 1, 8 with score 2, 14 with score 3, and 3 with score 4.
Qamar et al (2019)	60	52 (86.67%)	>10%	NR	Weak :20<br>Strong: 32	Staining was weak in 20 (38.46%) strong in 32 (61.5%) cases
Choi et al (2007)	60	NR	IHC score of 3.	Median 60.0% (range: 0.0~90.0%).	NR	None
Harshani et al (2014)	30	29 (96.67%)	>10	NR	Weak: 10 Moderate: 14 Strong: 5	• Normal tissue IHC staining score: 0 for 18 cases, 1 for 5 cases, 2 for 4 cases, 3 for 3 cases.<br>• OSCC tissue IHC staining score: 0 for 1 case, 1 for 10 cases, 2 for 14 cases, 3 for 5 cases
Angadi et al (2015)	30	30 (100%)	≥1%	25-50%: 16<br>>50%: 14	Mild: 10<br>Intense: 20	Expression was significantly greater in OSCC compared to healthy tissues.
Grimm et al (2014)	161	67 (42%)	≥10%	NR	NR	No GLUT-1 detected in normal samples
Wang et al (2017)	20	NR	Any brown staining of cells.	NR	NR	GLUT-1 in OSCC was significantly greater compared to normal samples, and was rarely detected in the latter.
Grönroos et al (2014)	5	4 (80%)	Presence of "slight netlike membrane staining"	Mean: 19% (0-60)	NR	None
Pereira et al (2016)	15	15 (100%)	NR	74.02% (54.2-86.2)	NR	None
Ohba et al (2010)	24	24 (100%)	>0%	NR	Weak: 13<br>Intense: 11	None
Oliver et al (2004)	54	49/54 (90.74%)	>0%	NR	Weak: 20<br>Moderate: 16	None

					Intense: 13	
Brands et al (2017)	15	12 (80%)	NR	NR	NR	No GLUT-1 detected in normal samples
Roh et al (2009)	42	30 (71.43)	≥1%	1–10%: 2 11–50%: 6 51–80%:14 >80%: 9	NR	None
Jensen et al (2015)	30	30 (100%)	NR	NR	Weak: 6 Moderate: 23 Strong: 1	None
Han et al (2012)	33	23 (70%)	≥1%	1-10%: 0 11-50%: 7 51-80%: 10 >80%: 6	NR	None
Miyawaki et al (2010)	37	30 (81%)	<0%	NR	NR	None
Xiao et al (2013)	14	12 (85.71%)	<0%	22.04% (0-100%)	NR	None
Kunkel et al (2003)	118	118 (100%)	<0%	NR	NR	None
