# Supplementary material for: The Role of Glucose Transporters in Oral Squamous Cell Carcinoma
Source: Biomolecules. 2021 Jul 21;11(8):1070. doi: 10.3390/biom11081070 (PMC8392467; doi:10.3390/biom11081070)
Supplement: Supplementary file 1 [file biomolecules-11-01070-s001.zip › Table S2 - Summary of findings for less studied GLUT family members.pdf]

**Table S2.** Summary of findings for less studied GLUT family members.

| Gene | Author | Tissue type | Modality | Findings |
|---|---|---|---|---|
| GLUT2 (*SLC2A2*) | Mellanen et al (1994)[48] | Tumour samples | mRNA | *SLC2A2* undetectable in 5/5 tumour samples |
| | Fukuzumi et al (2000)[38] | Cell lines | mRNA | *SLC2A2* expression in 5/9 OSCC cell lines and 2 normal epithelial lines |
| | Reisser et al (1999) [60] | Tumour samples | Protein | No GLUT2 expression in 1/1 tumour |
| GLUT8 (*SLC2A8*) | Deron et al (2011)[59] | Tumour samples | Protein | 35/35 tumours positive for GLUT8, mean H score 4.5 (SD: 3.7) |
| GLUT13 (*SLC2A13*) | Lee et al (2011)[91] | patient derived cell cultures | mRNA<br><br>protein | *SLC2A13* detected in 7/7 tumours with positive cells clustered together in islands within the tumour<br>*SLC2A13* increased in sphere forming cells over primary tumour cells<br><br>GLUT13 expression primarily on cell membrane |
| SGLT (*SLC5A*) | Helmke et al (2004)[92] | cell lines | mRNA | *SLC5A1* detected in 5/8 OSCC cell lines,<br>*SLC5A2* not detected |
| | Hanabata et al (2012)[39] | cell lines | protein | SGLT-1 detected in 6/6 OSCC cell lines |
